# Value-Based Framework for Evaluating Pre-Commercial Procurement: Case Study of Value-Based Key Performance Indicators

**DOI:** 10.2196/71279

**Published:** 2025-08-14

**Authors:** Nils-Otto Ørjasæter, Kari Jorunn Kværner, Linn Nathalie Støme

**Affiliations:** 1 Department of Early Psychosis, Oslo University Hospital, Forskningsveien 2B, 0373, Oslo, 0373, Norway, 47 91107841; 2 Department of Addiction Research, Oslo University Hospital, Oslo, Norway

**Keywords:** internet-based self-management system for treating chronic diseases, treatment of chronic patients, pre-commercial procurement of internet and digital-based solutions in health care, evaluation of pre-commercial procurement solutions, value-based procurement, value-based key performance indicators in health care, early health technology assessment

## Abstract

**Background:**

The demographic shift toward older populations is placing increasing pressure on health care systems, and only 20% of patients with chronic issues in the industrial world’s rural areas have guaranteed access to adequate health care services. This stresses the health care systems, emphasizing the need for innovative solutions. The Horizon 2020 Pre-Commercial Procurement (PCP) project, Crane, addresses these needs by facilitating the procurement of a digital self-management system for treating patients with chronic issues at home. Three rural European regions are participating in the project: Västerbotten (Sweden), Extremadura (Spain), and Agder (Norway).

**Objective:**

This study aims to explore and identify key design criteria and value-based key performance indicators (VB-KPIs) to support the development and evaluation of digital health care solutions for patients with chronic issues in rural areas within the Crane PCP process.

**Methods:**

A 3-iteration process was used to identify and prioritize the VB-KPIs in the Crane project. First, user needs were investigated based on stakeholder analyses in the participating rural regions. The early health technology assessment tool, Step Up, was used in 5 workshops (2 in Agder, 2 in Extremadura, and 1 in Västerbotten). Participants included patients and health care professionals. Second, post workshop, stakeholders were asked to comment on the summarized results, which were accordingly adjusted. Third, following the workshops, VB-KPIs were identified and prioritized, and discussions among representatives from the 3 buyer regions were conducted.

**Results:**

Thirty-five VB-KPIs across 5 domains were identified. User-related (9 VB-KPIs), employee-related (9 key performance indicators), clinical (4 VB-KPIs), organizational (6 VB-KPIs), and economic (8 VB-KPIs) outcomes from the workshops and the subsequent discussions emphasized regional differences in terms of user needs and priorities. While Agder (Norway) and Västerbotten (Sweden) emphasized privacy, digital trust, and physical interaction as important, Extremadura (Spain) prioritized negotiation and shared decision-making. Despite differences, shared values were identified, including empowerment, flexibility, preventative care, and improved quality of life.

**Conclusions:**

The identified and prioritized VB-KPIs are likely to provide a need-based foundation for the development and subsequent evaluation of the digital PCP, Crane, although regional socioeconomic and cultural differences may necessitate local adaptations.

## Introduction

### Background

Aging is one of the most important changes in population structure currently taking place in industrialized countries [1]. Increasing longevity and declining fertility rates are shifting the age distribution of populations in industrialized countries toward older age groups. As a result, the number of older adult people per 100 working-age people will nearly triple, from 20 in 1980 to 58 in 2060 [1]. The shift is more severe in rural areas than urban areas, mainly due to an increasing number of younger people moving to cities [1]. People living in rural areas face greater difficulties in accessing health and social care services. Older people require these services more frequently and often have additional challenges due to loss of mobility and cognitive function. It is assumed that around 37% of Europeans older than 65 years have multiple chronic conditions, although a 3-fold variation between EU member states is reported [2]. Older than the age of 65 years, men and women have an additional life expectancy of approximately 18 and 21 years, respectively. For both genders, only 9 of these years are expected to be of high quality of life. The remaining time is expected to represent age-related morbidity due to one or more chronic diseases (multimorbidity) [3].

In rural areas, great geographical distances and a lack of transportation services commonly represent additional challenges [4]. Thus, only 20% of patients with chronic issues in these areas can be guaranteed adequate health care services. The remaining 70%‐80% need to be empowered to increase self-management of health care needs [5]. These demographic changes are expected to stress the health care systems and urge the need for innovations. To meet these challenges, moving more of the health care to the patient’s home through digital solutions is a growing trend [6]. Crane [7] is a Horizon 2020 Pre-Commercial Procurement (PCP) project, compliant with the European Health Data Space [8] concept, addressing these challenges. PCPs enable the industry from the demand side to develop innovative solutions based on public sector needs. As such, the scheme provides a first customer reference that enables companies to create a competitive advantage in the marketplace. PCP allows public buyers to compare alternative potential solution approaches and filter the best possible solutions the market can offer to meet the public’s needs [7].

### The Basis for This Study

Crane is an internet and digital-based health care PCP project. The aim is to develop an integrated and flexible self-management system for treating patients with chronic issues at home, and thus improving the quality of life of the patients and optimizing the use of health resources.

Crane is run by partners from 3 rural regions: region Västerbotten in Sweden, Extremadura in Spain, and Agder in Norway. The project is divided into 3 phases. The first phase, “solution design,” was a feasibility study where 5 bidders were selected. In the second phase, “prototype development,” 3 proposals were selected from the first phase. This phase is still in progress, and the goal is to develop a prototype. Two suppliers will be selected in the third phase, “verification of prototype,” for field testing their proposed solution in 3 rural regions. This process allows us to use the participant rural areas as testing grounds to build sustainable health care solutions that can be exported to the rest of the world. One of the main goals of the PCP process is to lead to the development of commercial volumes of end-products in the European market.

An important part of the evaluation process, selecting the best solutions and bidders throughout the PCP process, is a value-based approach. Value-based procurement is increasingly becoming part of the innovation procurement processes [9]. This procurement process must focus not only on the price of a particular product or service, but also on the overall value of the solution it can create, in terms of improved patient outcomes, reduced total cost of care, and benefits to stakeholders such as health care workers [9].

Porter and Teisberg [10] divide value-based procurement into 4 dimensions. These dimensions highlight different aspects of the procurement. At the center of the specification are the two dimensions that (1) relate to patient outcomes and (2) relate to the cost of producing them. (3) The next dimension consists of other important aspects, with more secondary relevance to patient outcome, typically benefits for the health care personnel, the hospital, and the complete health care system. (4) The last dimension relates to societal impact, and how the purchase will impact society in a larger context, an aspect that is emphasized in the European public procurement directive [10].

### Aim of This Study

This study aims to explore and identify important design criteria for satisfying stakeholder needs in the PCP project, Crane. The aim of the study is 2-fold. First, to identify and prioritize value-based key performance indicators (VB-KPIs) to predict important positive and negative effects of the suggested PCP solutions. The VB-KPIs will be applied, prioritizing solutions from bidders throughout the PCP process. Second, the identification of regional differences in user needs. Other important key performance indicators (KPIs), such as evaluating technical compliance, capacity, and usability, were not part of this study.

## Methods

### Study Design

This study was reported according to the quality improvement reporting excellence (SQUIRE 2.0 [Standards for Quality Improvement Reporting Excellence]) publication guideline.

### Context

The participating European regions in the Crane project are Västerbotten, a rural region in the northern parts of Sweden, Extremadura, a region in the central-western parts of Spain, and Agder in the south of Norway. A needs assessment in the user regions identified medical areas of importance for the Crane efforts. The selected areas were the following: diabetes, mental health, chronic obstructive pulmonary disease (COPD), and cardiac disease. In Europe, these conditions account for at least 25% of health spending [2]. Stakeholder needs for the identification of VB-KPIs were provided by patients with chronic conditions, their next of kin, and health personnel in each region. Each region recruited patient representatives and health care personnel, and participation was voluntary.

We used a framework for early health technology assessment (eHTA) to identify the VB-KPIs among the stakeholders. eHTA evaluates technologies still in development and can be defined as “a health technology assessment conducted to inform decisions about 20 subsequent developments, research and/or investment by explicitly evaluating the potential value of a conceptual or actual health technology” [11]. Thus, eHTA is a suitable prioritization methodology in the early development stages before a solution is final.

We used the decision support eHTA framework Step Up [12] to identify and structure stakeholder needs in 4 domains:

- The user benefits: positive and negative implications the innovation will have for the patient, next of kin, and employees.
- The clinical effects: clinical values and risks of implementing the innovation.
- The organizational effects: the positive and negative implications the innovation will have for the operations locally and nationally.
- The economic effects: positive and negative financial implications the innovation will have locally and nationally in the short term and long term.

A coassessment and visualization tool was used to discuss potential effects among the users and stakeholders [12]. Potential effects within the 4 domains were rated on a scale from low to medium and high for each patient group or health condition identified by the stakeholders. The larger the imprint, the higher the potential benefits for the users and stakeholders.

### Intervention: Identifying VB-KPIs

Five workshops were conducted to identify VB-KPIs in the Crane project, 2 in Agder (Norway) and Extremadura (Spain) and 1 in Västerbotten (Sweden). Data collection of user needs was the focus of the workshops. The workshops were conducted digitally in Agder and Västerbotten and physically in Extremadura. Each workshop lasted approximately 2 hours and was organized with a facilitator and a secretary collecting and reporting the inputs. In the Agder workshop, with the 2 medical conditions, diabetes (10 participants) and mental health (9 participants), stakeholders were represented in 2 participating groups. The diabetes group consisted of 2 representatives of patients with diabetes, 1 diabetes coordinator, 1 physician, 1 nurse, and 5 other representatives from the health region. The mental health group consisted of 1 psychologist, 1 physician, 1 nurse, and 6 other representatives from the health region. In the Västerbotten workshop, COPD and cardiac diseases were represented. Present stakeholders were 2 nurses, 1 representative of patients with COPD, 2 representatives of patients with cardiac issues, and 2 other representatives from the health region. In Extremadura, 2 groups participated, 1 consisting of health care professionals and 1 with patient representatives. Both groups had 10 participants in total. The patient groups included 4 representatives of patients with diabetes and 4 representatives of patients with cardiac diabetes. The patient representatives were recruited by inviting various patient organizations within the selected chronic diseases in Crane. The participating patient representatives were the ones responding to the invitations. The participants in the workshops were recruited based on their connection to each chronic condition, not on the likelihood of engaging with the Crane solution in the future.

### Study of the Intervention and Measures

Before and at the start of the workshop, the participants were informed of the Crane goals and vision, and informed consent was given for participation in this study. Further, a future scenario was presented to the participants in every workshop to invoke discussions on potential effects: “What if digital solutions are in place fulfilling the Crane goals and vision, and the users have sufficient digital competence using the solutions?”

**Figure 1.**
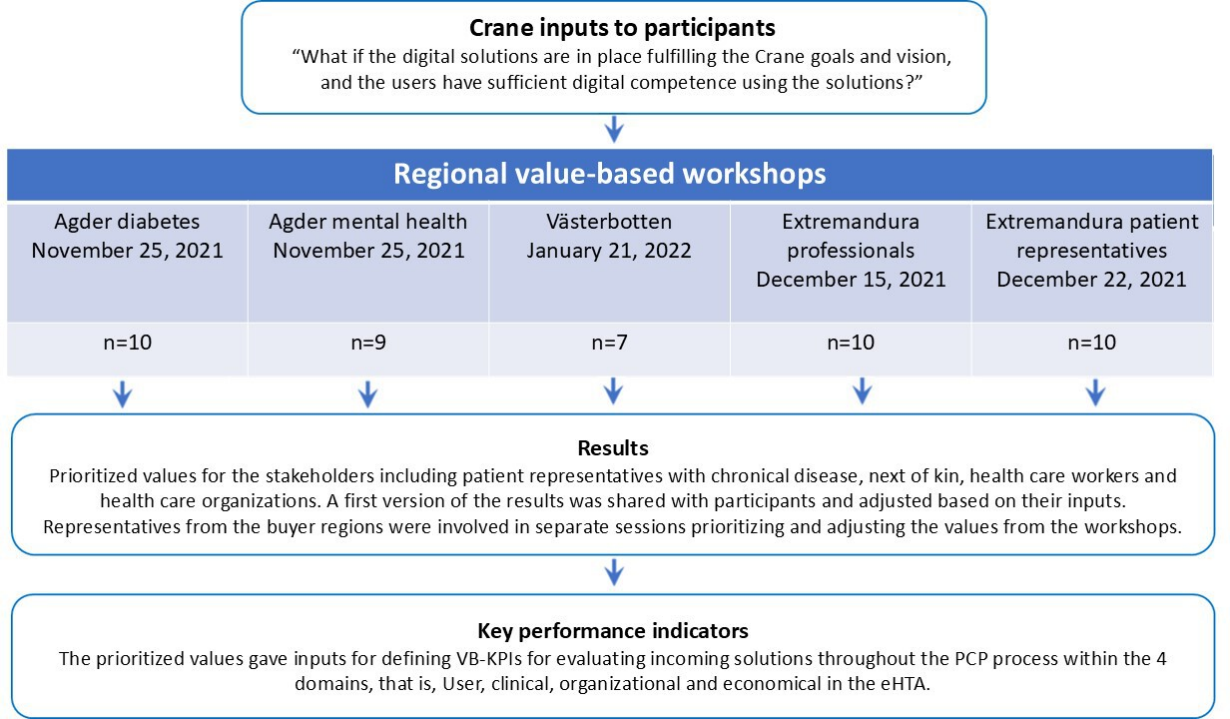
Identifying VB-KPIs in the Crane project. eHTA: early health technology assessment; PCP: Pre-Commercial Procurement; VB-KPI: value-based key performance indicator.

Potential positive and negative effects were identified and prioritized within the 4 domains given by the eHTA framework Step Up. These prioritized effects gave inputs on user needs and a basis for defining the VB-KPIs for evaluating incoming solutions throughout the PCP process. The process is visualized in more detail in Figure 1.

### Analysis

A process of 3 iterations was used to collect, analyze, and finalize the VB-KPIs in the Crane project. (1) The user needs identified by the stakeholders in the workshops were prioritized based on the eHTA Step Up framework [12] and (2) adjusted based on comments postworkshop. (3) In separate sessions with the buyer regions, the VB-KPIs were then identified and prioritized based on stakeholder needs.

VB-KPIs were collected and structured within the 4 domains of the framework and simplified by merging patients and next of kin in the user domain. Local and national effects were merged in the economic domain. The organizational domain was simplified to positive and negative effects for the health care organization. The clinical domain was simplified to positive and negative health effects for the patient.

### Ethical Considerations

This study is not subject to approval from the Regional Committees for Medical and Health Research Ethics (Norway), as it does not contain medical and health research on human beings, human biological material, or personal health data. This is not research on humans according to the definition in the Norwegian Health Research Act § 4 [13]. The study did not process special categories of personal data according to GDPR (General Data Protection Regulation), article 9, number 1 [14]. Participation in the 5 workshops was voluntary. Collected data from the workshops were based on informed consent and handled following GDPR. The legal basis for processing data is consent according to GDPR, article 6, number 1a. The informed consent form was given to the participants when invited to the workshop. The information was also given at the start of the workshops, and no reservations were given. Collected data from the 5 workshops were written down by referees in their native languages, anonymized, translated to English, and transferred in an encrypted key to the researchers at Oslo University Hospital. The recorded and transferred data were never linked to the informant, ensuring anonymity. No compensations were given to the participants.

The collected data are only personal data according to GDPR article 6. Processing of personal data followed the principles in GDPR article 5. The data were anonymized and transferred properly and securely. The specific situation of the data subject has been taken into consideration since there were representatives from patient organizations. They were invited as representatives from organizations, not as patients in a health care situation. The asymmetry is taken into consideration since the representatives are voluntary delegates in the organization, and the project is outside the health and medical care situation.

## Results

### Identified Needs

The workshops gave important insights into the potential positive and negative effects of implementing the Crane solution within the 4 domains.

Structuring the inputs from the workshops in all regions showed potential positive and negative effects within the following main areas: (1) increase in efficiency, (2) needs for ensuring safety, control, and trust in data sharing, (3) needs for knowledge, innovation, and research, (4) increased needs for interaction and networking, and (5) needs for considering the state of mind, attitude, and worries.

Mapping these main topics with the different domains gave overall results for all regions. When an iteration process with the stakeholders was applied to achieve consensus on the implications of Crane, the first iteration results from the workshops were shared with the stakeholders to provide inputs and comments. The result, including inputs and comments, is shown below (Tables1  2). Complete tables of the results from the workshops are available in MultimediaAppendices 1  2.

**Table 1.** Stakeholders’ needs in efficiency, safety, and knowledge versus the 4 domains (user, clinical, organizational, and economic).

Domains	Efficiency	Safety, control, and trust	Knowledge, innovation, and research
User			
Positive	Handling of the disease Increased flexibility Less burden, traveling, use of medicine, hospitalizations, and consultations Increased time for support and follow-up Availability of information	Better overview control, awareness, and feeling of safety Fast response to changes and acute situations Recognition from health care workers and relatives	Empowerment of self-care Increased knowledge and awareness New type of patient contacts
Negative	No input	Privacy issues and loss of trust Information overload and complexity	Wrong use of the equipment Technological literacy
Clinical			
Positive	Fewer incidents, adverse events, and complications Better follow-up and infection control Right treatment at the right time	Better control of the disease and the treatment Less setbacks Increased quality of life	Learn from earlier actions Potential preventive actions
Negative	Increase in measuring parameters Measurement failures Cyber attacks Technology-related problems	Less physical contact Technology and data focus Too much standardization of treatment	Lack of competencies
Organizational			
Positive	Decrease in cancelations and work overload Shorter treatment queues Better allocation of resources More consultations Reduced demand for consultation per patient Use of experts	Increased quality of the health care service Better documentation Better routines and follow-up	No inputs
Negative	Increase in measurements Implementation and scale-up challenges Change management and transition Interpretation of monitoring data Increase in alarms and emergencies	Increase in measurements Operating the follow-up system; routines and processes Data management and GDPR^a^ compliance Cybersecurity issues	New training and educational activities
Economic			
Positive	Decrease in cancelations Fewer hospital admissions More patients per health care worker Easier and faster handling of patients Less need for hospital space Less travel costs	Environment-friendly	Research activity Knowledge and training
Negative	Investments and funding Cost of training patients and caregivers Compensation challenges	Increased need and cost of cybersecurity and GDPR issues Increased system costs	Increased training and education costs

a GDPR: General Data Protection Regulation.

**Table 2.** Stakeholders’ needs in interaction and networking, and state of mind, attitude, and worries versus the 4 domains (user, clinical, organizational, and economic).

Domains	Interaction and networking	State of mind, attitude, and worries
User		
Positive	Easier access to health care and health care workers Maintain contact even in bad times Less disturbance from health care workers entering at home Create networks across country borders Discuss how to improve the health parameters	Increased self-esteem More freedom Information about the times and indicators in which you have been monitored More exciting New ways of working
Negative	Still important with physical interactions between patients and health care workers Empathic treatment must not be underestimated There is a need for long-term relations Less physical contact and the ability to see the complete picture Interaction between systems	Stress issues when looking at data Information overload Increased feeling of loneliness “Burnout” See that their values are becoming worse Difficult to use for patients who are very sick Negative influence from others
Clinical		
Positive	No inputs	Better control of the disease and the treatment Less setbacks Increased quality of life
Negative	No inputs	Less physical contact Technology and data focus Too much standardization of treatment
Organizational		
Positive	Easier to work across disciplines Better flow of data between health care actors Closer interactions with patients	More equal health care
Negative	Many actors and interfaces to deal with Third-party dependencies Two systems: physical contact and digital interaction Centralization: a system spanning over a larger geographical area may lose out on local and geographical context	New ways of working Will be confronted with ethical questions that are not seen today Possible moral dilemmas among professionals
Economic		
Positive	Opening for interaction between technology and people	No inputs
Negative	No inputs	Possible abuse of evidence

While Agder and Västerbotten emphasized quite similar effects, Extremadura differed from the other 2 regions on the following topics:

1. Interaction and networking: none of the groups in Extremadura emphasized positive effects of interaction and networking; rather, the stakeholders in Extremadura weighted negotiations and shared decision-making between the patient and health care personnel. The groups from Agder and Västerbotten stressed the need for interaction between patients, experts, and health care organizations.
2. Privacy and GDPR compliance [14]: Agder and Västerbotten both emphasized the importance of safeguarding privacy and GDPR compliance, while this topic was not mentioned by Extremadura.
3. Hacking and cyber-attack issues were stressed by the groups in Agder and Västerbotten but not by Extremadura.
4. Physical presence for health care workers to see the overall picture and reduce the patient’s feeling of loneliness. The need for still having physical meetings between patients and the health care workers was emphasized by the Agder and Västerbotten groups, but not by the Extremadura groups.

Some subtopics seemed to be important to all groups:

1. Quality of life: improved well-being and treatment quality
2. Empowerment of self-care and involvement of the patient and next of kin
3. Awareness: improved self-control
4. Preventive care: improvement in preventive care opportunities
5. Flexibility and freedom for patients, health care workers, and their organizations
6. Excitement: health care workers carrying out new health care pathways and achieving new knowledge; patients and next of kin getting better insights and control
7. Stress: health care workers receiving information overload, resulting in a new work reality; patients and next of kin feeling pressure to relate to the data output, getting alarms, and more responsibility

These are topics that should be implemented in the evaluation and selection of the solution throughout the PCP process.

### Scoring of the Health Value Domains

**Figure 2.**
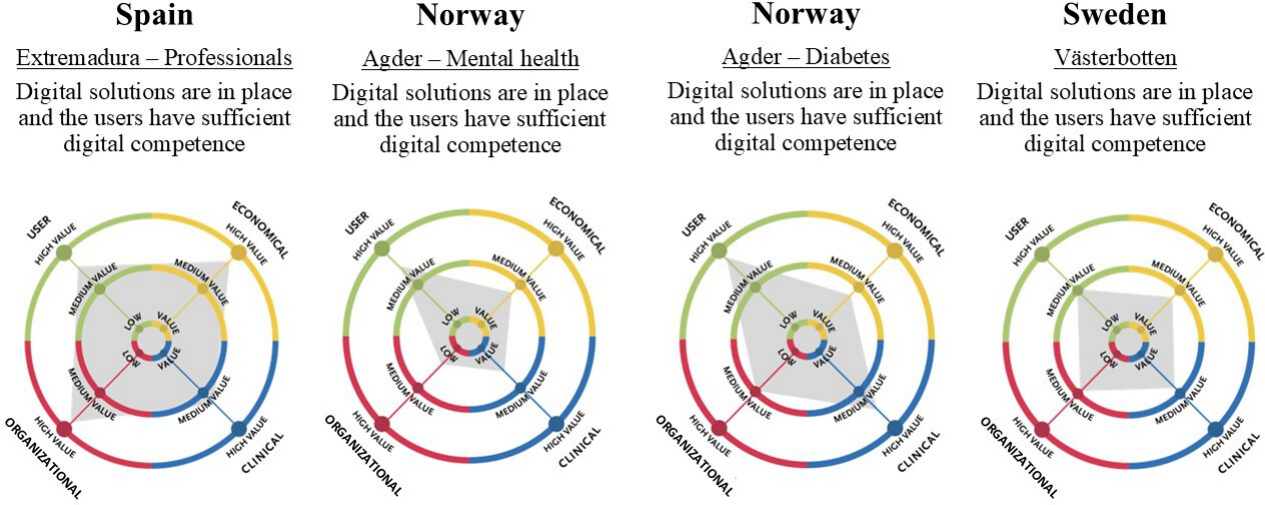
The different regions and groups scored the domains using the Step Up coassessment tool.

The second iteration consisted of the rating within the 4 health value domains. The stakeholders from the workshops rated a potential value of the Crane solution, from 0 low value to 100 high value, using the eHTA framework, Step Up [12]. The results are shown in Figure 2 and Table 3. In Extremadura, only the professional group rated the potential of the future scenario.

The different regions’ and groups’ scoring of the domains. The scores from the health value spider are presented in the table (0, low-100, or high).

The user, economic, and organizational domains were assumed to be high in Extremadura. The clinical domain was predicted to be more moderate. In Agder, both the mental health and the diabetes groups scored the user domain as high and the economic domain as moderate. The clinical domain was assumed to be high for the patients with diabetes and low for the patients with mental health issues. In Västerbotten, all the domains were assumed to be moderate. The scoring was used as input in discussions of prioritizing domains and needs and used as input in defining VB-KPIs by the buyer regions in the third iteration.

**Table 3.** The different regions and groups’ scoring of the domains.

Regions	Groups	Domains			
		User	Economic	Organizational	Clinical
Extremadura	Professionals	80	88	90	66
Agder	Mental health	75	45	20	25
Agder	Diabetes	90	45	45	70
Västerbotten	Mixed	50	45	50	45

### Values and KPIs

In the third and final iteration, the VB-KPIs were identified and prioritized. To actualize the identified needs in the VB-KPIs, analyses were performed by involving representatives from the buyer regions in separate sessions after the workshops. Discussions within the workshops and follow-up by professionals from the buyer regions resulted in a prioritized list of 35 potential needs (effects) to be considered in the evaluation process (Tables4  5).

**Table 4.** Patient and employee–related VB-KPIs^a^ were identified and prioritized by the buyer regions based on values identified and scored in the stakeholder workshops.

User domain		Values	KPIs^b^ to be considered
Patients			
	1	Increased feeling of loneliness	Perceived degree of loneliness
	2	Improves the empowerment of self-care	Perceived level of self-control of the situation
	3	Relatives obtain insight into their loved one’s health	Perceived relatives’ insight into the patient
	4	Creates a higher level of trust in sharing data	Perceived level of trust in sharing data
	5	Creates an improved well-being	Perceived level of well-being
	6	Fast response to changes when it is an acute situation	Average response time in acute situations per patient per year
	7	Easier access to health care workers and the health care system in a hectic daily life	Perceived level of accessibility of health care workers
	8	Increased flexibility	Perceived level of flexibility
	9	Avoid stress	Perceived level of stress
Employee			
	10	Timesaving	Average (or perceived) use of time per patient per month
	11	More patients for follow-up	Average (or perceived) number of patients per month per health care worker
	12	Fast response	Average time from request to respond per month
	13	Good overview	Perceived a better overview of the patient’s health condition
	14	Helps in decision-making by knowing the patient profile more accurately	Perceived ability to make better decisions for the patients
	15	Continued contact with patients	Average number of contacts per patient per month
	16	Increased flexibility	Perceived level of flexibility
	17	Increased stress	Perceived level of stress
	18	More exciting work conditions	Perceived as more exciting work conditions

a VB-KPI: value-based key performance indicator.

b KPI: key performance indicator.

**Table 5.** Economic, organizational, and clinical VB-KPIs^a^ identified and prioritized by the buyer regions based on values identified and scored in the stakeholder workshops.

Domains		Values	KPIs^b^ to be considered
Economic			
	19	Cost of damages and repair	Total cost of repair, that is, IT^c^ and equipment per month
	20	Training and education costs, and time used for training and interpretation of results from the patients	Total cost of education and training, health care workers, and patients handling the solution
	21	Less hospitalization	Average hospitalization days per year per patient
	22	Less use of medicine	Average amount of medicine used per patient per month
	23	Less physical consultations	Average number of physical consultations with patients per month
	24	Lower travel costs for patients and health care workers	Average travel cost due to treatment and consultations for patients and health care workers
	25	Investments in the solution and support	Investment and cost calculations
Organizational			
	26	Decrease in cancelations	Average number of various types of cancelations per month
	27	Shorter treatment queues	Average time before treatment takes place per group of patients
	28	Increase in alarms and emergencies	Average number of alarms and emergencies per patient per month
	29	Better services and professional standards	Perceived quality of the service and professional standards
	30	Data management and GDPR^d^ compliance	Evaluation of compliance and fulfillment of standards
	31	Better cooperation between health care services and actors	Perceived level of cooperation between the organization and other actors
Clinical			
	32	Better control of one’s health and fewer setbacks	Average QALY^e^, that is, EQ-5D
	33	Less focus on the complete health condition of the patient due to fewer physical contacts	Perceived change of focus, seeing the complete condition
	34	Better well-being and quality of life	Average QALY, that is, EQ-5D
	35	Fewer health risks and complications	Average number of alarms and emergencies per patient per month

a VB-KPI: value-based key performance indicator.

b KPI: key performance indicator.

c IT: information technology.

d GDPR: General Data Protection Regulation.

e QALY: quality-adjusted life year.

## Discussion

### Principal Findings

This study aimed to explore and identify key design criteria and VB-KPIs to support the development and evaluation of digital health care solutions for patients with chronic issues in rural areas within the Crane PCP process. The European rural regions of Västerbotten (Sweden), Extremadura (Spain), and Agder (Norway) are the project partners in Crane. A process of 3 iterations of stakeholder insight was used to identify and prioritize the VB-KPIs based on the eHTA framework Step Up. We identified 35 VB-KPIs across 5 domains. Despite differences in user needs, shared values were found, including empowerment, flexibility, preventative care, and improved quality of life. However, based on our findings, regional socioeconomic and cultural differences may necessitate local adaptations in the final solution in Crane.

Although we successfully identified a set of cross-national VB-KPIs to support the procurement process of Crane, these results should be applied with caution, considering the local context. One limitation to the transferring of the VB-KPIs between regions is the different prioritization of domains (Figure 2 and Table 3) and user needs identified by the participating regions in the project. Even though the 3 regions (Agder, Västerbotten, and Extremadura) had several similar characteristics and were defined as rural regions, there were geographical, cultural, and socioeconomic differences that became visible in the needs specification of the VB-KPIs. Needs that differed between Norway and Sweden compared with Spain were privacy and security, interaction between patient and health care personnel, and the need for physical interaction with patients. An analysis performed by the Cultural Group in 2024 [15] pointed to cultural differences between Spain and the northern countries. Spain scores lower on preferences for individualism, long-term orientation, and indulgence, and higher on dimensions such as power distance, motivation toward achievement and success, and uncertainty avoidance [15]. As for socioeconomic differences, the gross domestic product per capita in 2023 was US $ 90.50 in Norway, US $ 64.19 in Sweden, and US $ 46.35 in Spain [16]. Based on statistics from the European Commission from 2021, the 3 countries each spent between 10%‐11% of their gross domestic product on health care expenditures [17]. These differences will most likely influence the health care system and the patient’s priorities, reactions, and willingness to adopt a service such as Crane. Thus, when planning for PCP projects crossing national borders, both cultural and socioeconomic differences should be addressed, and local adjustments to the final solution should be made accordingly.

### Further Research

An important next step is the verification of the selected VB-KPIs by the stakeholders. In the next phase of the project, the tenders will be evaluated according to the identified VB-KPIs from this study. After the evaluation and prioritization of the tenders, the selected solutions will undergo field testing and piloting, and patients and health care workers will test the solutions in their local environments. The data collection from these feasibility tests will provide valid input in the final selection of the solution and a basis for regional adjustments. This makes room for continuous adjustments such that the final solution may comply with predefined and shifting user needs, that is, using an agile approach. As stated by Holbeche [18], the ability to continuously adjust and adapt (agility) is increasingly considered to be a vital success factor for organizations. This coincides with the formative evaluations of eHTA, where an innovation is evaluated while still under development.

Further research classifying KPIs for different types of PCP projects is a needed contribution to the selection of the most viable PCP solutions.

Broadening the scope by including other geographical factors, socioeconomic differences, and adjustments based on age groups in the VB-KPIs should be an area of further research.

### Limitations

The outcomes from this study may not be generalized, as the VB-KPIs are retrieved from workshops and discussions with a limited number of participants and user groups. This affects the validity of this study, which may be strengthened by adjusting the needs and VB-KPIs based on continuous evaluations after each tender phase of the PCP process. Further, information on socioeconomic and geographical differences between the participating regions should be included. This may, for instance, entail variation in distance from the respective rural areas to more densely populated areas where satisfactory follow-up is available. Another limitation was that the stakeholder groups also differed in the health conditions they focused on in each region. Each workshop was also influenced by the personal interests and personalities of the stakeholders. Further, although 4 chronic disease conditions were represented, no information from other dominant chronic disorders in the regions was included. An important strength, however, is that the participants represented different European countries, thus increasing the relevance of the solution in a broader European context.

### Conclusions

Although adjustments are needed to fit the specific aims of the given PCP project, the recommended VB-KPIs may represent a substantial contribution when evaluating digital PCP projects within health care. The identified and prioritized VB-KPIs provide a need-based foundation for the development and subsequent evaluation of Crane. The use of a structured framework based on eHTA provides an opportunity to verify the identified effects through economic modeling as the Crane solution develops, to ensure that user needs are met. One significant finding in the early assessment of stakeholder needs was that, although shared values were identified across nations in the rural regions, the need for local adjustments to the final solution should always be addressed.

## Supplementary material

10.2196/71279 Multimedia Appendix 1 Complete output from the stakeholder workshops: efficiency, safety, control, and trust, and knowledge, innovation, and research.

10.2196/71279 Multimedia Appendix 2 Complete output from the stakeholder workshops: interaction and networking, and state of mind, attitude, and worries.

## Abbreviations

COPD: chronic obstructive pulmonary disease
eHTA: early health technology assessment
GDPR: General Data Protection Regulation
KPI: key performance indicator
PCP: Pre-Commercial Procurement
SQUIRE: Standards for Quality Improvement Reporting Excellence
VB-KPI: value-based key performance indicator

## References

[1] Jones K (2020). The problem of an aging global population, shown by country. Visual Capitalist.

[2] Introduction to healthcare systems. European Federation of Pharmaceutical Industries and Associations.

[3] Brennan P, Perola M, van Ommen GJ, Riboli E, European Cohort Consortium (2017). Chronic disease research in Europe and the need for integrated population cohorts. Eur J Epidemiol.

[4] (2008). Poverty and social exclusion in rural areas: final study report. https://hdl.handle.net/11380/606205.

[5] (2024). CRANE – Pre-Commercial Procurement Project.

[6] Digital health. World Health Organization.

[7] (2024). Pre-Commercial Procurement. European Commission, Research and Innovation.

[8] (2024). European Health Data Space Regulation (EHDS). European Commission.

[9] (2024). How procurement unlocks value-based health care. Boston Consulting Group (BCG).

[10] (2017). Value-Based Procurement (VBP): Knowledge, Guide, and Support All in the Value Chain of Medical Technology.

[11] Grutters JPC, Bouttell J, Abrishami P (2025). Defining early health technology assessment: building consensus using Delphi technique. Int J Technol Assess Health Care.

[12] Kværner KJ, Støme LN, Romm J (2021). Coassessment framework to identify person-centred unmet needs in stroke rehabilitation: a case report in Norway. BMJ Innov.

[13] (2008). The Norwegian Health Research Act § 4. https://app.uio.no/ub/ujur/oversatte-lover/data/lov-20080620-044-eng.pdf.

[14] (2024). General Data Protection Regulation (GDPR). GDPR.eu.

[15] (2024). Country comparison tool. The Culture Factor Group.

[16] (2024). GDP per capita, consumption per capita and price level indices. Eurostat.

[17] Healthcare expenditure statistics – overview. Eurostat.

[18] Holbeche L (2023). The Agile Organization: How to Build an Engaged, Innovative and Resilient Business.

